# The Action of Belladonna

**Published:** 1869-03

**Authors:** 


THE following conclusions, in reference to the action of Belladonna arrived at by M. Meuriot, can not but be of interest to our readers.—ED.

I. " Atropine is the active principle of belladonna, and assumes all the properties of this solanum.

II. The intensity of its action varies with the species of animals. Herbivora are less sensible to its action than the Carnivora. In man its poisonous action is the most violent; but no animal is exempt.

III. Its action also varies with the dose employed; for small doses accelerate the heart's pulsation and augment the vascular tension; poisonous doses diminish the tension and modify the cardiac pulsations.

IV. Belladonna is a vasculo-cardiac poison, in the classification of M. Sée. Its action produces especially the innervation of the heart and of the vessels.

V. The varied phenomena produced by Atropine depend mostly upon its primordial and elective action, or are due to the elimination of the poison.

VI. Atropine acts upon the heart through the pneumogastric nerve, whose peripheral extremities are paralyzed. It augments the frequency of the cardiac pulsations.

VII. In a small dose it augments the tonicity of the vascular muscles; in a poisonous dose it diminishes, and even destroys this; whence the application of Belladonna to epilepsy, in which the access seems to be due to modifications of cerebral circulation.

VIII. The variations of the arterial tension are subordinate to the state of excitation or paralysis of the muscular coat of the vessels.

IX. In small doses atropine accelerates the respiration; in poisonous doses it diminishes its frequency.

The acceleration of these movements depends upon the excitation of the respiratory centers; the consecutive retardation, upon a paralysis of the extremities of the vagi nerves; whence its application in the treatment of asthma.

X. Atropine in a therapeutical dose, increases the activity of the excito-motory functions of the spinal cord.

In a poisonous dose it exagerates the reflex power till it may produce convulsions.

XI. Atropine *always* produces agitation, insomnia, delirium, and in a poisonous dose, coma; it is not at all a narcotic.

XII. Atropine is eliminated by the kidneys, by all the mucous surfaces, and sometimes by the skin of man. Its elimination is always rapid; so that the action is of short duration.

XIII. The effects due to elimination are numerous, viz.: redness of the mucous surfaces and of the skin, frequent desire of micturition; colic; anal and vesical tenesmus; profuse sweat, diarrhœa, etc.

XIV. The redness and dryness of the mucous membrane explains aphonia, dysphagia, dysuria, etc.

XV. Not only are all the secretions of the mucous membrane diminished, but there may be also, on account of the activity of the circulation, a rapid reabsorption of all the liquids which have exuded from mucous surfaces or from wounds; whence its advantage in exaggerated secretions, and its effect upon coughs, etc.

XVI. Atropine, applied locally to the tissues, produces an activity of the capillary circulation, and, in a considerable dose, true hyperæmia and sanguineous stasis.

Angina and erythema produced by belladonna are analogous to the inflammatory process.

XVII. The modification of the urinary secretions are dependent upon the variations of the arterial tension.

XVIII. Belladonna is not a paralyzing agent to the smooth muscular fibres; it produces no phenomena of paralysis except in a very powerful dose, and in those cases it follows exaggerated contractions; thus it is of benefit in incontinence of urine and of the fæces, in paralysis of the bladder, in constipation, irreducible hernias, etc.

XIX. Atropine has no elective action upon the sensitive nerves. Its local application is always followed by acute and persistent pain. Atropine acts only upon nerves in a state of hyperæsthesia and often determines analgesia, but it should be applied directly upon the seat of pain (les nerfs affectés).

XX. Small doses of atropine augment, toxical doses diminish, the temperature.

XXI. Atropine, especially, possesses the property of causing dilatation of the pupil, and this is its most constant and persistent effect.

It paralyzes the terminal branches of the third pair of nerves; this is the only fact well shown by experimental physiology, in the study of the hyoscyamus also. (Gubler, Mydriasis caused by Belladonna.

To this paralysis of the ciliary branches of the nervous motor ocularis communis is attached the paralysis of the muscle of accommodation.

XXII. Certain experiments and several considerations that have been made public by the author, tend to show some exciting action upon the sympathetic nerve or upon the *dilatateur*. However, a more vigorous demonstration is still essential.

